# The incidence of retinopathy of prematurity in neonates in Germany in 2019; a nationwide epidemiological cohort study

**DOI:** 10.1007/s00431-023-05229-0

**Published:** 2023-11-30

**Authors:** Ahmad Samir Alfaar, Melih Parlak, Omneya Hassanain, Eman Abdelmaksoud, Armin Wolf

**Affiliations:** 1 Department of Ophthalmology, Ulm University Hospital, Ulm, Germany; 2 International Medical Neuroscience, Ophthalmology, Charité Medical University, Mittelalee 4, Augustenburger Platz 1, 13353 Berlin, Germany; 3 St. Paul Eye Unit, The Royal Liverpool University Hospital, Liverpool, UK; 4 Clinical Research Department, Children’s Cancer Hospital Egypt (CCHE-57357), Cairo, Egypt; 5 Pediatrics Department, Saint John Regional Hospital, Saint John, New Brunswick, Canada

**Keywords:** Retinopathy of prematurity, Incidence, Risk factors, Treatment, Mortality

## Abstract

Retinopathy of prematurity (ROP) is a leading cause of childhood blindness in preterm infants. The incidence of ROP varies widely across countries, with rates as high as 30% in some regions. This study investigated the incidence, risk factors, treatment, and mortality of ROP patients in Germany. Data were extracted from the German Federal Statistical Office (Destatis) diagnosis-related group (DRG) and Institute for the Remuneration System in Hospitals (InEK) databases. Patients with a secondary diagnosis of ROP (ICD-10 code H35.1) in the first 28 days of life were included. Data were extracted for patients admitted between January 1, 2019 and December 31, 2019. The diagnoses and procedures were determined using the German version of the International Classification of Diseases (ICD-10-GM) and the German procedure coding system (OPS). The codes 5–154.xx, 5–155.xx, 8–020.xx, 5–156.9, 6–003.(c&d), 6–007.(2&8) were utilised to denote different ocular treatments. Patient Clinical Complexity Levels were extracted and used to compare ROP with non-ROP patients. A total of 1326 patients with ROP were identified. The incidence of ROP is estimated to be 17.04 per 10,000 live births. The incidence was highest in infants with birth weights less than 500 g and decreased with increasing birth weight. The most common risk factors for ROP were low birth weight, male sex, and prematurity. Of the infants with ROP, 7.2% required ocular treatment. The most common treatment was intraocular injections, followed by photocoagulation. No surgical treatment was required for any of the infants during the study period. The mortality rate for infants with ROP was 60.33 per 10,000. This is higher than the overall neonatal death rate of 24.2 per 10,000.

*Conclusions*: This study found that the incidence of ROP in Germany is similar to that in other developed countries. The study also found that the mortality rate for infants with ROP is higher than the overall neonatal death rate. These findings highlight the importance of early detection and treatment of ROP in preterm infants.

**Table:** 

**What is Known:** *• ROP is a severe eye condition often affecting preterm infants.* *• Previous data are limited in scope and generalizability.*	
**What is New:** *• Based on a national database, our study found ROP incidence to be 17.04 per 10,000 new births, higher in males (17.71) than in females (16.34).* *• 7.2% of ROP cases required ocular treatment, inversely correlated with birth weight.* *• High rates of multimorbidity such as neonatal jaundice (84.69%), respiratory distress syndrome (80.84%), and apnea (78.88%) were observed.*

**Supplementary Information:**

The online version contains supplementary material available at 10.1007/s00431-023-05229-0.

## Introduction

Retinopathy of prematurity (ROP) is a retinal disease that affects preterm neonates who need admission to the neonatal intensive care unit due to one of several morbidities, including extremely low birth weight and the associated underdevelopment of the lungs, sepsis, and other afflictions [1, 2]. The retinal vascularisation starts to develop around the 16^th^ week of gestation and has been found to reach full maturation around the 40^th^ week of gestation [3]. Retinal vasculature is expectedly underdeveloped in premature babies, and the resultant relatively high oxygen flow leads to dysregulation of the vascular endothelial growth factor (VEGF). Consequently, retinal metabolism increases, and in the presence of poor vascularisation, retinal hypoxia and detachment may ensue [4–6].

ROP typically develops in five stages, each with its specific management [7]. Neonatal screening for ROP is generally based on qualifying risk factors identified by the pediatricians, who then refer patients to an experienced ophthalmologist [8]. Therefore, paediatricians and general practitioners must be able to identify key risk factors and make proper referrals.

Albeit several registers are currently being established, most current studies concerning ROP have either been conducted as single-center screening studies or as collaborative studies between hospitals [9, 10]. Scarce data are available on the incidence and comorbidities of the ROP determined through population-based studies. This has resulted in a lack of sufficient information required for proper resource planning and population-based quality management.

Our study aimed to estimate the national incidence of ROP as reported in the first month of life and investigate the associated morbidities in Germany during 2019. Moreover, to compare the comorbidities in the patients with ROP to those that did not develop ROP.

## Methods

### Population and study design

This cohort study identifies and analyses the patients within a defined population, adhering to the STROBE guidelines for accurate and comprehensive reporting of observational studies.

### Data sources and extraction

Data were extracted from the Diagnosis-related group (DRG) statistics database of the German Federal Statistical Office (Statistisches Bundesamt (Destatis)), utilised for secondary data analysis. In compliance with the Hospital Remuneration Act (KHEntgG, Sect. 21), the data was retrieved via the G-DRG browser hosted by the Institute for the Remuneration System in Hospitals (InEK), accessed on 16.09.2021 and revised on 25.06.2023.

### Inclusion and exclusion criteria

**Fig. 1:**
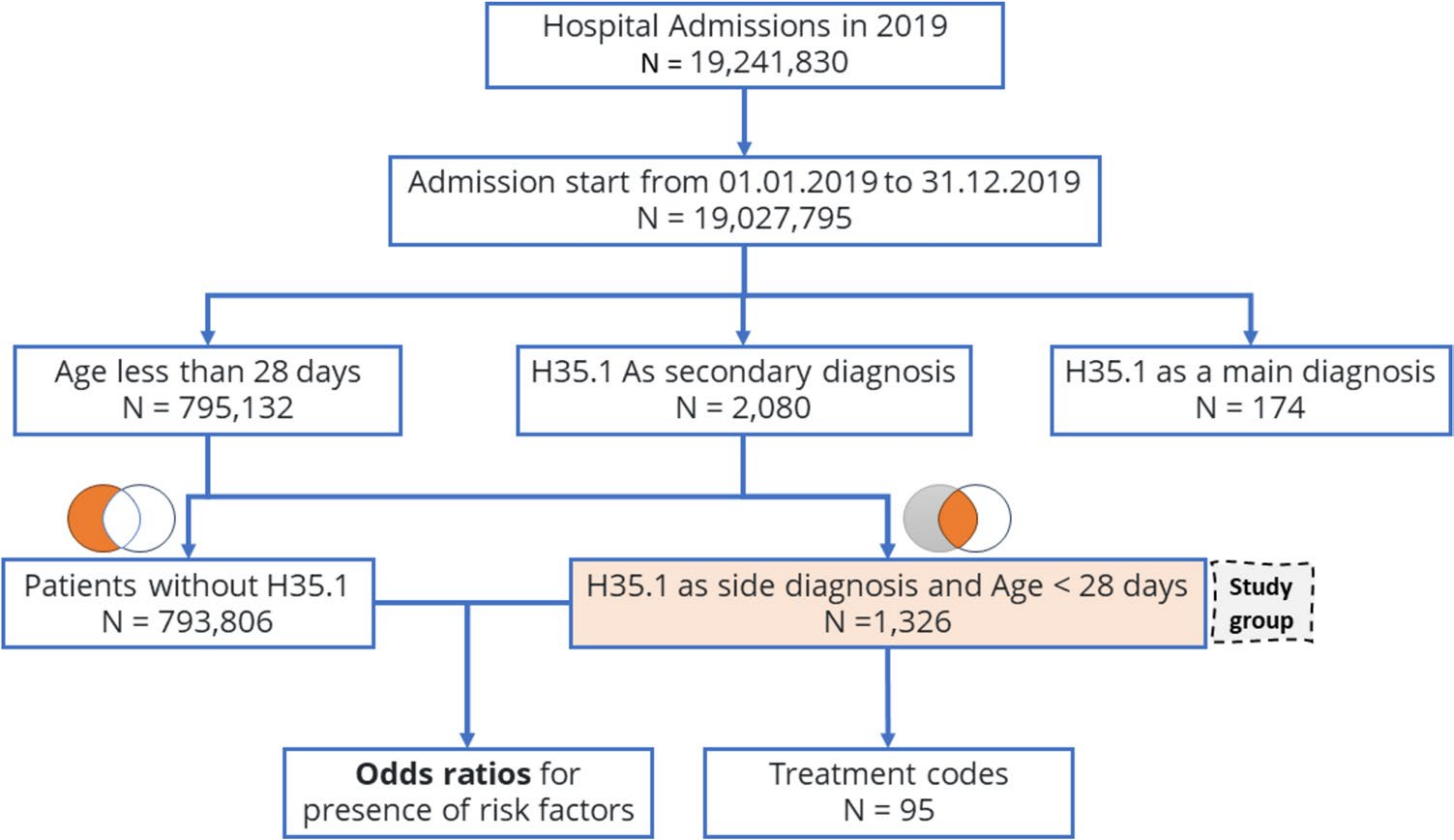
Flow chart of patients’ inclusion in the study

Patients included in the study were those with a secondary diagnosis of Retinopathy of Prematurity (ROP, ICD-10 code H35.1) identified within the first 28 days of life. The selection process favored patients treated for sequelae of prematurity or during screening post-delivery in maternity or pediatric wards from January 1, 2019 to December 31, 2019. Cases excluded were those beyond 28 days at the time of diagnosis or admitted outside the study period (Fig. 1).

### Data variables

The research further involved extracting multiple datasets, including neonatal admissions data — particularly emphasising those requiring intensive care—and neonatal mortality data. Diagnostic and procedural codes (e.g., 5–154.xx, 5–155.xx) as per the German variation of the ICD-10 and the national procedure coding system delineated various ocular treatments. The Patient Clinical Complexity Levels (PCCL) were also sourced to comprehensively compare ROP and non-ROP patients.

### Ethical considerations

Upholding the principles outlined in the Declaration of Helsinki and the International Conference on Harmonisation (ICH), the study secured the ethical sanctity of the process. Given the German authorities’ public availability of anonymised data, IRB approval was not necessitated.

### Statistical analysis

Aligning with STROBE guidelines, the statistical analysis embraced a structured approach. Patient data was synthesised, presenting numbers and percentages. A keen analysis on the mean length of hospital stay and its standard deviation facilitated the calculation of the Homogeneity coefficient (HC) as a representation of data homogeneity [11, 12]. Incidence rates were projected per 10,000 cases, incorporating a distinct death rate analysis based on weight categories to determine early death risk in extremely low birth weight infants, further predicting ROP incidence rate assuming survival.

The odds ratio (OR) computations followed the methodology that Altman (1991) illustrated, facilitating comparative analysis of neonates with and without reported ROP [13–15]. This analysis utilised Microsoft® Excel® (version 16.0.14326.20164) and Google Sheets, ensuring the privacy of groups with four or fewer patients by not disclosing specific details.

## Results

### Incidence and demographics

In 2019, out of approximately 778,090 new births in Germany, which equated to 795,132 neonatal admissions, 1326 were diagnosed with ROP, representing a rate of 17.04 per 10,000 new births. Notably, males (53.32%, *n* = 707) were slightly more affected than females (46.68%, *n* = 619), with incidence rates of 17.71 and 16.34 per 10,000, respectively (Table 1).

**Table 1:** Patients’ characteristic

Characteristic			
Total	1326 patients		
Sex		N	%
	Male	707	53.32%
	Female	619	46.68%
Duration of stay (in days)	Mean	79.5	
	Standard deviation	33.7	
	Homogeniety cooeficient	70.21%	
Duration of stay according to standard		N	%
	Short	4	0.30
	Normal	914	68.93
	Long	408	30.77
Birth weight	Birth weight	Cases	%
	< 500	89	6.7
	500– < 750	337	25.4
	750– < 1000	336	25.3
	1000– < 1250	203	15.3
	1250– < 1500	130	9.8
	1500– < 2500	102	7.7
	Unknown	129	9.7

*N *number of patients

### Hospital admissions and diagnoses

**Fig. 2:**
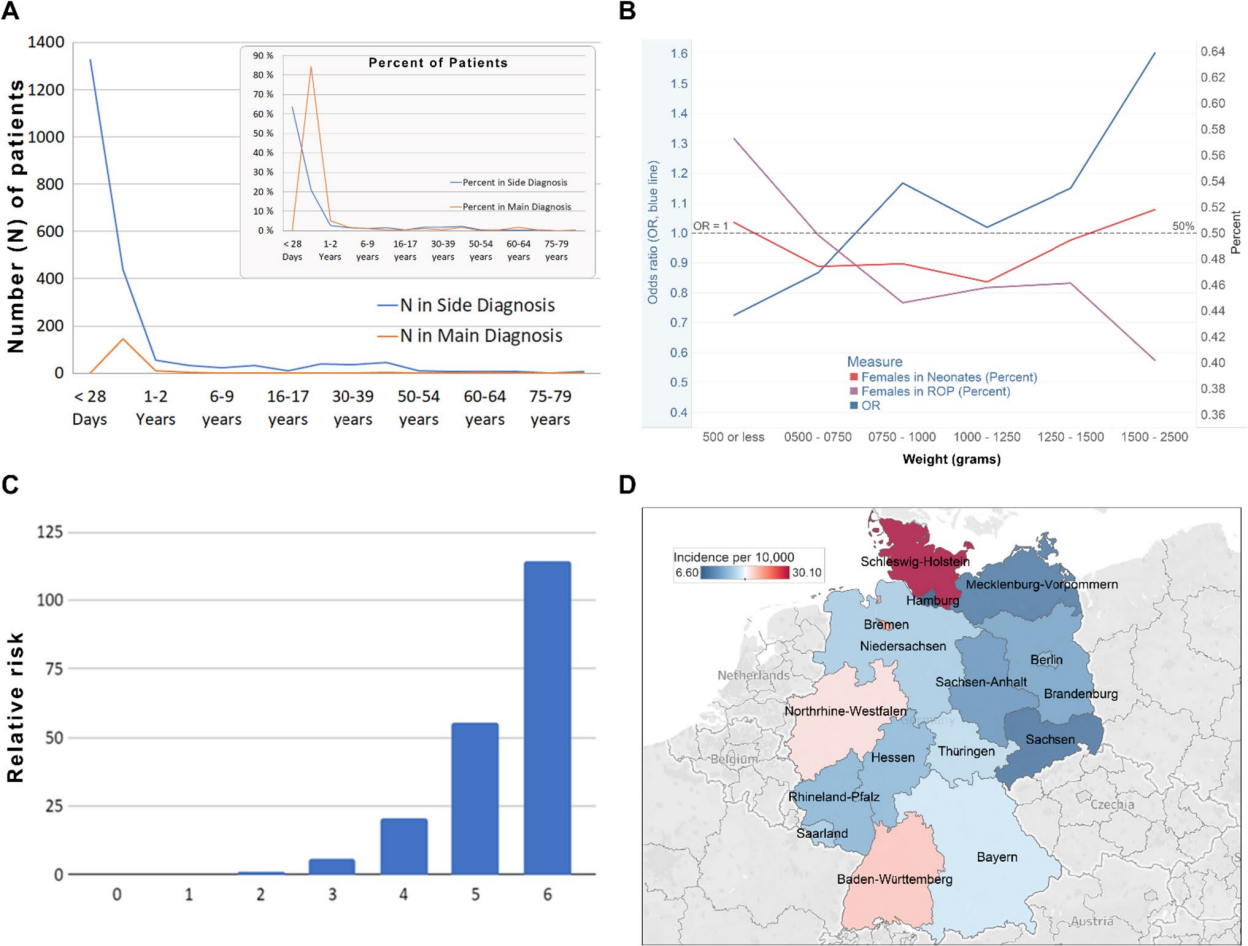
Patients’ characteristics **A** Age distributions of Retinopathy of Prematurity (ROP) patients according to numbers and percent. **B** Percentage of females and association with weight. **C** The relative risk (Y-Axis) of having a Patient-Related comorbidity Score in ROP (X-Axis). **D** Rate of diagnosis of ROP among newborns per state

During the initial admission period, ROP primarily presented as a secondary diagnosis, with no instances noted as the primary reason. A significant decrease in secondary diagnosis admissions was observed from day 28 to 1 year (*n* = 438), while the primary diagnosis admissions peaked at 147 patients. The trend stabilised beyond this period (Fig. 2a).

### Analysis based on birth weight

The data delineated the incidence of ROP according to various birth weight categories. A staggering 90.27% of ROP patients had a principal diagnosis of low birth weight, with significant representation in the 500–750 g and 750–1000 g categories (25.41% and 25.34%, respectively) (Table 1, Supplementary Table 1). An intriguing pattern was noticed in the female population within the ROP group, displaying a step-wise decrease with the incremental increase in birth weight, revealing a significant OR of 1.60 (95% CI 1.08–2.38) in the 1500–2500 g category (Fig. 2b).

### Duration of hospital stay

The mean hospital stay duration was notably prolonged, averaging 79.5 days (SD = 33.7), significantly higher than the general neonatal population (4.2 days, SD = 7.1). The length of stay demonstrated an inverse relation to birth weight, ranging from 119.6 days (for ≤ 500 g) to 38.3 days (for 1500–2500 g).

### Comorbidities

Patients tended to multimorbidity, with a marked increase in the relative risk of ROP with escalating comorbidity scores (Table 2, Fig. 2c). Predominant comorbidities included neonatal jaundice (84.69%), respiratory distress syndrome (80.84%), apnea (78.88%), and anaemia of prematurity (71.42%), among others (Supplementary Table 2). Remarkably, certain comorbidities were more prevalent in specific birth weight groups, indicating potential associations that necessitate further exploration (Supplementary Table 3).

**Table 2:** Patients-Related Comorbidity Scores (PCCL)

**PCCL**	**N in ROP**	**% in ROP**	**N In non-ROP**	**% in non-ROP**	**OR (95% CI)**	***p*-value**
0	105	7.9%	747,592	94.2%	0.01 (0.00–0.01)	0.0001
1	0	0.0%	94	0.0%	3.2 (0.20–51.1)	0.4158
2	6	0.5%	3440	0.4%	1.04 (0.5–2.3)	0.9158
3	292	22.0%	29,499	3.7%	7.3 (6.4–8.3)	0.0001
4	287	21.6%	8119	1.0%	25.9 (22.7–29.5)	0.0001
5	426	32.1%	4174	0.5%	81.3 (72.2–91.6)	0.0001
6	210	15.8%	888	0.1%	136.1 (116.1–159.5)	0.0001

*PCCL *Patients-Related Comorbidity Scores, *ROP *Retinopathy of Prematurity, *N *count, *Other n *other in neonates, *OR *odds ratio

### Ocular treatment

Ocular interventions were necessary in 7.2% of cases, predominantly involving intraocular injections (50.5%), with Ranibizumab being the most commonly used drug (58.3%). An evident inverse correlation existed between the necessity for ocular treatments and birth weight (Supplementary Table 1).

### Geographical distribution

A noticeable variation was observed in the Diagnosis rates of ROP across different German states, hinting at potential regional influences affecting the prevalence. Schleswig–Holstein reported the highest rate (30.1 per 10,000), whereas Hamburg had the lowest, necessitating further investigative efforts to understand the underlying causes (Fig. 2d).

### Mortality

Eight patients succumbed during the admission period, translating to a heightened death rate of 60.33 per 10,000 compared to the general neonatal death rate of 24.2 per 10,000.

## Discussion

The prevalence of Retinopathy of Prematurity (ROP), a critical eye disease observed in premature infants, has been substantially influenced by advancements in neonatal care since the 1940s. This study elucidates various factors influencing the incidence and management of ROP in neonates, drawing upon a rich pool of data from German hospitals and paralleling international studies and guidelines.

### Global and regional perspectives on ROP

ROP has been recognised as a significant challenge in neonatal healthcare, with its incidence being intimately tied to the improved survival rates of preterm babies due to enhanced neonatal care practices. Despite the significant strides made globally in reducing ROP-induced blindness, disparities exist, with some regions witnessing a surge in ROP cases concomitant with rising neonatal survival rates [16]. This scenario emphasises the necessity for optimised screening protocols and interventions, particularly in regions grappling with this increasing burden.

### Screening protocols and guidelines

Understanding the variability in screening protocols internationally is essential in delineating a comprehensive approach to ROP management. Infants with a weight under 1.5 kg are commonly the target group for ROP screening, though the exact criteria can differ based on several factors, including the level of development in a country and the specifics of individual neonatal courses [17–19].

The American Academy of Pediatrics (AAP), the American Association for Pediatric Ophthalmology and Strabismus (AAPOS), and the American Academy of Ophthalmology (AAO) propose stringent guidelines for ROP screening, particularly emphasising the necessity to screen infants with certain risk factors, such as low birth weight and prolonged oxygen support [19]. Concurrently, German guidelines rely heavily on the ETROP study's recommendations, emphasising a more nuanced approach based on gestational age and oxygen administration duration [20–23].

### Incidence and associated risk factors

Reaching the actual incidence rates, it is discerned that the previously reported rates often stem from collaborative efforts of expert centers, and not from an epidemiological background, potentially portraying a skewed representation of the disease spectrum. Our study, which covered the whole German population, delineated specific associated factors, underlining the critical role of birth weight and unveiling the near-universal incidence of ROP in neonates with a weight less than 750 g. This observation hints at potential underreporting or overlooking of ROP in certain NICUs, a prospect warranting further investigation to uncover other contributing factors possibly dampening the reported incidence rates.

### Gender disparities in ROP incidence

An intriguing facet of our study was the exploration of gender-specific variations in ROP occurrence. Despite the majority of literature concurring on the non-significant role of gender in ROP development, our study highlighted an increasing trend of ROP in larger birth weight females. This observation necessitates a deeper analysis, considering the potential influence of sex hormones on vascular development and the differential responses to stressful perinatal conditions exhibited by different genders [24, 25].

### Technological advancements and network integration

In the face of mounting challenges, including the scarcity of specialised ophthalmologists and the proliferation of neonatal centers, a shift towards telemedicine approaches seems not only viable but indispensable. Incorporating non-invasive ocular imaging techniques and developing remote consultation platforms are paving the way for a more integrated and efficient approach towards ROP management [26–33].

Moreover, ROP management’s success is increasingly attributed to cohesive networks involving various healthcare professionals. These networks foster adherence to established guidelines, promoting quality and consistency in ROP care [34].

### Current treatment modalities

The treatment landscape for ROP has witnessed significant transformations, with the advent of anti-VEGF therapy marking a significant milestone. Our study emphasised the efficacy of this intervention, echoing the promising results demonstrated in recent studies [35–37]. However, the pursuit for more robust evidence continues, necessitating further studies with a comprehensive design to substantiate the preliminary findings.

### Generalizability

The findings of this study, based on an extensive analysis of data from the German DRG database, primarily offer insights into neonatal ROP cases within the German healthcare context between January and December 2019. While the results showcase notable trends and relationships, the generalizability may be limited due to the study’s geographical and temporal scope. Therefore, caution should be exercised when attempting to apply these findings to different healthcare systems, time frames, or broader populations. Future research should aim to expand the scope to include multi-national datasets for a more comprehensive understanding and global applicability of the results.

### Study limitations and future directions

While our study offers a significant contribution to the analysis of ROP incidence in Germany, it has several constraints that must be considered. First, the lack of detailed clinical data impedes the possibility of performing a multivariate analysis, potentially affecting the depth and breadth of our findings. This limitation highlights a significant gap, as a more comprehensive dataset would permit nuanced insights into the underlying trends and relationships in ROP cases.

Second, the statistical tools employed in this research did not sufficiently address the data’s heterogeneity. Specifically, the absence of measures such as the Gini coefficient (GSI) hindered a fuller understanding of population distribution and risk factors associated with ROP, limiting the depth of the analysis.

Recognising these constraints, future research should prioritise incorporating a broader spectrum of statistical methods capable of facilitating a multi-variable analysis, fostering a more detailed examination of data heterogeneity. Additionally, merging robust clinical data with statistical analysis is paramount. This integration would not only fill the existing gaps in the current study but also forge pathways to developing data-driven, context-specific interventions that could significantly enhance ROP prevention and management strategies. By addressing these areas, we can aim to provide a more comprehensive and nuanced understanding of ROP incidence globally, thereby improving the precision and effectiveness of future preventative strategies.

### Supplementary Information

Below is the link to the electronic supplementary material.

Supplementary file1 (DOCX 26 KB)

Supplementary file2 (DOCX 33 KB)

## Data Availability

Datasets related to this article can be found at https://www.g-drg.de/inek-datenportal, an open-source online data repository hosted by the Institute for the Remuneration System in Hospitals.

## Abbreviations

DRG: Diagnosis-related group
HC: Homogeneity coefficient
OR: Odds ratio
PCCL: Patient Clinical Complexity Levels
ROP: Retinopathy of Prematurity
