# Supplementary material for: The incidence of retinopathy of prematurity in neonates in Germany in 2019; a nationwide epidemiological cohort study
Source: Eur J Pediatr. 2023 Nov 30;183(2):827–34. doi: 10.1007/s00431-023-05229-0 (PMC10912137; doi:10.1007/s00431-023-05229-0)
Supplement: Supplementary file 1 — Supplementary file1 (DOCX 26 KB) [file 431_2023_5229_MOESM1_ESM.docx]

Supplementary Table 1. Main diagnosis for admission of ROP patients and risk against those without ROP

| Code | Main Diagnosis | N | % | (N) Other Neonates | Inc*% | OR ** | Therapy (N) |
| --- | --- | --- | --- | --- | --- | --- | --- |
| P07.00 | New-borns: birth weight less than 500 grams | 89 | 6.7 | 387 | 18.7 | 147.8 ( 317.7 - 423.1) | 18 |
| P07.01 | New-borns: birth weight 500 to less than 750 grams | 337 | 25.4 | 738 | 31.3 | 366.6 ( 200.1 - 262.8) | 40 |
| P07.02 | New-borns: birth weight 750 to less than 1000 grams | 336 | 25.3 | 1175 | 22.2 | 229.3 ( 78.7 - 107.9) | 14 |
| P07.10 | New-borns: birth weight 1000 to less than 1250 grams | 203 | 15.3 | 1557 | 11.5 | 92.1 ( 28.2 - 40.8) | 5 |
| P07.11 | New-borns: birth weight 1250 to less than 1500 grams | 130 | 9.8 | 2544 | 4.9 | 33.9 (1.8 - 2.7) |  |
| P07.12 | New-borns: birth weight 1500 to less than 2500 grams | 102 | 7.7 | 29410 | 0.3 | 2.2 (8.6 - 16.8) |  |
| P22.0 | Respiratory distress syndrome of the Newborn | 36 | 2.7 | 1840 | 1.9 | 12 (1.4 - 4.1) | < 4 |
| P28.5 | Respiratory failure in the Newborn | 14 | 1.1 | 3534 | 0.4 | 2.4 (22.6 - 69.9) | < 4 |
| P07.2 | Newborn with extreme immaturity | 13 | 1 | 198 | 6.2 | 39.8 (0.2 - 0.7) | < 4 |
| P07.3 | Others born before the appointment | 10 | 0.8 | 16032 | 0.1 | 0.4 | < 4 |
|  | Others | 56 |  | 737717 |  | 0 | < 4 |
|  | Total | 1326 |  | 795132 |  | 0 | 95 |

ROP: Retinopathy of Prematurity, N: Count, * Incidence among neonates of that group in percent, ** p <0,0001 except P28.5 and P07.3 p<0,002, *** p <0,0001 except P28.5 p<0,002 and P07.3 p>0,5

Supplementary Table 2. Secondary Diagnoses in the patients with ROP (Sorted by odds ratio, OR, p< 0.0001)

| Code | Secondary Diagnosis | N | % | N in other Neonates | OR (95%CI ) |
| --- | --- | --- | --- | --- | --- |
| E89.0 | Hypothyroidism after medical measures | 4 | 0.3% | 1 | 2405.8 (268.7 - 21539.9) |
| Z27.4 | Need for measles-mumps-rubella vaccination [MMR] | 5 | 0.4% | 2 | 1504.8 (291.7 - 7763.2) |
| M85.80 | Other specified changes in bone density and structure: Multiple locations | 6 | 0.5% | 3 | 1204.7 (301.0 - 4822.2) |
| D61.9 | Aplastic anemia, unspecified | 4 | 0.30% | 3 | 801.9 (179.3 - 3586.7) |
| P27.1 | Bronchopulmonary dysplasia originating in the perinatal period | 424 | 32.0% | 481 | 776.2 (670.8 - 898.2) |
| Z99.0 | Dependence (long-term) on the aspirator | 6 | 0.5% | 5 | 722.8 (220.3 - 2371.4) |
| Y59.9 | Complications from vaccines or biologically active substances | 24 | 1.8% | 23 | 637.2 (358.8 - 1131.9) |
| T81.1 | Shock during or as a result of a procedure, not elsewhere classified | 4 | 0.3% | 4 | 601.5 (150.3 - 2407.5) |
| E55.0 | Florid rickets | 4 | 0.3% | 5 | 481.2 (129.1 - 1793.9) |
| E83.59 | Calcium metabolism disorders, unspecified | 4 | 0.3% | 5 | 481.2 (129.1 - 1793.9) |
| R73.0 | Abnormal glucose tolerance test | 4 | 0.3% | 5 | 481.2 (129.1 - 1793.9) |
| B25.88 | Other cytomegaly | 11 | 0.8% | 15 | 443.4 (203.3 - 967.2) |
| B25.9 | Cytomegaly, unspecified | 8 | 0.6% | 11 | 438.7 (176.2 - 1092.5) |
| Z23.8 | Need for immunization against other single bacterial diseases | 494 | 37.3% | 1146 | 411.1 (362.6 - 466.1) |
| K22.3 | Perforation of the esophagus | 4 | 0.3% | 6 | 401 (113.0 - 1422.6) |
| Z27.8 | Need for vaccination against other combinations of infectious diseases | 412 | 31.1% | 928 | 385.6 (337.6 - 440.4) |
| P07.2 | Extreme immaturity of New-born | 566 | 42.7% | 1544 | 382.5 (339.4 - 431.2) |
| Z27.3 | Need for vaccination against diphtheria-pertussis-tetanus with poliomyelitis [DPT + polio] | 223 | 16.8% | 458 | 350.7 (295.7 - 415.9) |
| P22.0 | Respiratory distress syndrome of New-born. | 1072 | 80.8% | 9472 | 349.6 (304.5 - 401.4) |
| K91.4 | Dysfunction after colostomy or enterostomy | 17 | 1.28% | 31 | 333.1 (183.9 - 603.3) |

Supplementary Table 3. Secondary Diagnoses of ROP patients with a birthweight of 1500-2500 (p<0.0001)

| Code | Secondary diagnosis | N | % | N in Non-ROP | OR ( 95%CI) |
| --- | --- | --- | --- | --- | --- |
| P91.2 | Cerebral leucomalacia in the newborn | 4 | 3.92% | 36 | 33.46 ( 11.7 - 95.8) |
| Z27.8 | Need for vaccination against other combinations of infectious diseases | 5 | 4.90% | 53 | 28.7 ( 11.2 - 73.4) |
| Z25.8 | Need for vaccination against other specified individual viral diseases | 8 | 7.84% | 121 | 20.8 ( 9.9 - 43.7) |
| P61.2 | Anemia with prematurity | 60 | 58.82% | 2308 | 18.3 ( 12.3 - 27.2) |
| P59.0 | Neonatal jaundice associated with premature birth | 79 | 77.45% | 6837 | 14.8 ( 9.3 - 23.6) |
| K40.90 | Inguinal hernia, unilateral or without a side indication, without entrapment and without gangrene: Not referred to as a recurrent hernia | 5 | 4.90% | 110 | 13.8 ( 5.5 - 34.6) |
| P22.0 | Respiratory distress syndrome of the newborn | 60 | 58.82% | 3331 | 12.7 ( 8.5 - 18.8) |
| Z22.8 | Carriers of other infectious diseases | 7 | 6.86% | 172 | 12.6 ( 5.8 - 27.6) |
| P03.6 | Damage to the fetus and newborn from abnormal uterine contractions | 4 | 3.92% | 97 | 12.4 ( 4.5 - 34.4) |
| R01.1 | Heart murmur, unspecified | 4 | 3.92% | 99 | 12.2 ( 4.4 - 33.7) |
| P83.3 | Other unspecified edema specific to the fetus and the newborn | 5 | 4.90% | 131 | 11.6 ( 4.7 - 3) |
| P02.7 | Damage to the fetus and neonate from chorioamnionitis | 13 | 12.75% | 394 | 10.9 ( 6.1 - 19.8) |
| P37.8 | Other specified congenital infectious and parasitic diseases | 7 | 6.86% | 202 | 10.8 ( 4.9 - 23.5) |
| P29.3 | Persistent fetal circulation | 8 | 7.84% | 236 | 10.6 ( 5.1 - 22.2) |
| Z29.1 | Immune prophylaxis | 4 | 3.92% | 121 | 9.96 ( 3.6 - 27.5) |
| P28.2 | Seizures of cyanosis in the newborn | 7 | 6.86% | 231 | 9.4 ( 4.3 - 20.5) |
| Q25.6 | Pulmonary artery stenosis (congenital) | 4 | 3.92% | 144 | 8.4 ( 3.0 - 23.0) |
| P28.4 | Other newborn apnea | 64 | 62.75% | 6063 | 8.2 ( 5.5 - 12.3) |
| P74.3 | Disorders of the potassium balance in the newborn | 10 | 9.80% | 387 | 8.3 ( 4.3 - 16.1) |
| P07.3 | Others born before the appointment | 82 | 80.39% | 15186 | 8.0 ( 4.9 - 1) |
| P71.1 | Other neonatal hypocalcaemia | 7 | 6.86% | 329 | 6.6 ( 3.1 - 14.4) |
| P74.2 | Disorders of the sodium balance in the newborn | 12 | 11.76% | 601 | 6.6 ( 3.6 - 12.0) |
| B96.2 | Escherichia coli [E. coli] and other enterobacteriacees as a cause of diseases that are classified in other chapters | 18 | 17.65% | 1076 | 5.9 ( 3.5 - 9.8) |
| Q21.1 | Atrial septal defect | 12 | 11.76% | 808 | 4.9 ( 2.7 - 8.9) |
| P28.5 | Respiratory failure in the newborn | 40 | 39.22% | 4042 | 4.7 ( 3.2 - 7.0) |
| P61.1 | Polyglobules in the newborn | 7 | 6.86% | 482 | 4.5 ( 2.1 - 9.8) |
| I95.8 | Other hypotension | 8 | 7.84% | 588 | 4.2 ( 2.1 - 8.8) |
| 92.2 | Reluctance to drink in the newborn(feeding difficulties) | 60 | 58.82% | 10246 | 4.1 ( 2.8 – 6.1) |
